# Numerical and Non-Asymptotic Analysis of Elias’s and Peres’s Extractors with Finite Input Sequences †

**DOI:** 10.3390/e20100729

**Published:** 2018-09-23

**Authors:** Amonrat Prasitsupparote, Norio Konno, Junji Shikata

**Affiliations:** 1 Graduate School of Environment and Information Sciences, Yokohama National University, Yokohama 240-8501, Japan; 2 Department of Applied Mathematics, Faculty of Engineering, Yokohama National University, Yokohama 240-8501, Japan

**Keywords:** true random number generation, von Neumann’s extractor, Peres’s extractor, Elias’s extractor

## Abstract

Many cryptographic systems require random numbers, and the use of weak random numbers leads to insecure systems. In the modern world, there are several techniques for generating random numbers, of which the most fundamental and important methods are deterministic extractors proposed by von Neumann, Elias, and Peres. Elias’s extractor achieves the optimal rate (i.e., information-theoretic upper bound) h(p) if the block size tends to infinity, where h(·) is the binary entropy function and *p* is the probability that each bit of input sequences occurs. Peres’s extractor achieves the optimal rate h(p) if the length of the input and the number of iterations tend to infinity. Previous research related to both extractors has made no reference to practical aspects including running time and memory size with finite input sequences. In this paper, based on some heuristics, we derive a lower bound on the maximum redundancy of Peres’s extractor, and we show that Elias’s extractor is better than Peres’s extractor in terms of the maximum redundancy (or the rates) if we do not pay attention to the time complexity or space complexity. In addition, we perform numerical and non-asymptotic analysis of both extractors with a finite input sequence with any biased probability under the same environments. To do so, we implemented both extractors on a general PC and simple environments. Our empirical results show that Peres’s extractor is much better than Elias’s extractor for given finite input sequences under a very similar running time. As a consequence, Peres’s extractor would be more suitable to generate uniformly random sequences in practice in applications such as cryptographic systems.

## 1. Introduction

Many cryptographic systems require random numbers, and the use of weak random numbers leads to insecure systems. In fact, many past security problems were due to the use of weak random numbers [1,2,3,4]. This tells us that random number generation is very important in cryptography, in particular to ensure that secret keys are random and unpredictable. In the modern world, there are several techniques for generating random numbers. A natural source such as physical phenomena, the stock market, or Bitcoin [5] can produce unpredictable random sequences, although such sequences are not uniformly random at the source (i.e., biased). However, there is a solution to solve this problem, namely, to use deterministic extractors. A deterministic extractor is a function which takes a non-uniformly random sequence as input and outputs a uniformly random sequence. The deterministic extractors have been studied in mathematics, information theory, and cryptography. In information theory, those extractors can also be treated for the intrinsic randomness problem (i.e., the problem of generating truly random numbers). Furthermore, as applications in cryptography, the output sequence of those extractors can be used as secret keys in information-theoretic cryptography or symmetric key cryptography. The extractors by von Neumann [6], Elias [7], and Peres [8] are fundamental and important ones. In particular, Elias’s and Peres’s extractors are interesting, since they can achieve the optimal rate (or redundancy), if we suppose that input size tends to infinity (i.e., from an asymptotic viewpoint). However, it is not easy to conclude which one is better, since those are constructed by completely different approaches. The main purpose of this paper is to investigate those with finite inputs (i.e., from a non-asymptotic viewpoint) by numerical analysis to make it clear which is better for practical use.

### 1.1. Related Work

Several works have proposed methods for extracting uniform random sequences from non-uniform random sequences. The most famous among them is von Neumann’s extractor [6] proposed in 1951. He demonstrated a simple procedure for extracting independent unbiased bits from a sequence of independent, identically distributed (i.i.d.) and biased bits. The technique by von Neumann guarantees that the output sequences are independent and uniform if the input sequence is independent and constantly biased, while this cannot be guaranteed if the bias is not constant (e.g., see [9]).

An improved algorithm of von Neumann’s extractor was proposed by Elias [7] in 1971. Elias’s extractor utilizes a block coding technique to improve the rate (or redundancy) of von Neumann’s extractor; however, the straightforward implementation of this extractor requires exponential time and exponential memory size with respect to *N*, where *N* is the block size, to store all $2^{N}$ input sequences with their assignment of output sequences. In 2000, Ryabko and Matchikina [10] proposed an extension of Elias’s extractor that improved time complexity and space complexity by using the enumerative encoding technique from [11] and the Schönhage–Strassen algorithm [12] for fast integer multiplication in order to compute the assignment of output sequences. In this paper, we call this improved method the RM method.

Peres’s extractor is another extended algorithm of von Neumann’s extractor. In 1992, Peres [8] proposed a procedure which improved upon von Neumann’s extractor. The basic idea of Peres’s extractor is to reuse the discarded bits in von Neumann’s extractor by iterating similar procedures in von Neumann’s extractor.

The extractors by von Neumann, Elias, and Peres are the most fundamental and important ones using a single source. In particular, Elias’s and Peres’s extractors are interesting, since they can achieve the optimal rate (i.e., information-theoretic upper bound) h(p) if input size tends to infinity (i.e., in an asymptotic case), where each bit of input sequences from a single source occurs with probability p∈(0,1) and h(·) is the binary entropy function. In this paper, we are interested in the non-asymptotic case, namely, the achievable rate for finite input-sizes. The rate of Elias’s extractor for finite input-sizes can be observed in the work [7], but the rate of Peres’s extractor for finite input-sizes is not explicitly known. As a work related to Peres’s extractor, Pae [13] reported a recursion formula to compute the rate for finite input-sizes, but it is difficult to give the rate function with finite input-sizes since the recursion formula is complicated. Pae also computed the rate by the recursion formula in the case p=1/3, compared the rates of Peres’s extractor and Elias’s extractor, and concluded that the rate of Peres’s extractor increased much slower than that of Elias’s extractor via numerical analysis. However, it is not explicitly known which extractor is better to use in practice, if we take into account the running time, implementation cost, and memory size required in the extractors, as mentioned in [13].

There are several works for constructing extractors using multiple sources (i.e., not a single source). Bourgain [14] provided a 2-source extractor under the condition that the two sources are independent and each source has min-entropy 0.499n, where *n* is the bit-length of the output of the sources. Raz [15] proposed an improvement in terms of total min-entropy, and constructed 2-source extractors with the condition that one source has min-entropy more than n/2 and the other source requires min-entropy O(logn). In 2015, Cohen [16] constructed a 3-source extractor, where one source has min-entropy δn, the second source has min-entropy O(logn) and the third source has min-entropy O(loglogn). In 2016, Chattopadhyay and Zuckerman [17] proposed a general 2-source extractor, where each source has a polylogarithmic min-entropy. They combined two weak random sequences into a single sequence by using K-Ramsey graphs and resilient functions. Their extractor has only one-bit output and achieves negligible error and higher complexity than Peres’s extractor or Elias’s extractor.

Furthermore, there are various reports about extracting random bits in the real world. In particular, in 2009, Bouda et al. [18] used mobile phones or pocket computers to generate random data that is close to truly random data. They took 12 pictures per second then used their function to obtain four random bits in each picture, and then applied Carter–Wegman universal${}_{2}$ hash functions. Halprin and Naor [19] presented the idea of using human game-play as a randomness source in 2009. They constructed the Hide and Seek game that produced approximately 17 bits of raw data per click, and then generated with a pairwise independent hash function a 128-bit string which is $2^{64}$-close to the random one in less than two minutes. In 2011, Voris et al. [20] investigated the use of accelerators on the RFID tags as a source. They implemented a two-stage extractor on the RFID tags. It can produce 128 random bits in 1.5 s by storing a Toeplitz matrix on the RFID tags and performing matrix multiplications.

### 1.2. Our Contribution

In this paper, we revisit the extractors by von Neumann, Elias, and Peres, since they are fundamental and only require a single source. In the studies on those extractors, it is normal to asymptotically analyze the rate or redundancy of the extractors in the literature, where the rate is the average bit-length of outputs per bit of input (see Section 2 for details). Specifically, the rate of von Neumann’s extractor is p(1−p) that is far from the optimal rate (i.e., information-theoretic upper bound) h(p). Meanwhile, the rate of Elias’s extractor converges to h(p) if the block size tends to infinity. Specifically, Elias’s extractor outputs a uniformly random sequence at a high rate, when it takes a long block size equal to the input length. However, it has a trade-off between the rates and computational resources such as time complexity and memory size. On the other hand, Peres’s extractor achieves the optimal rate h(p) if the length of input and the number of iterations tend to infinity, and it requires smaller time complexity and memory size. However, it would be hard to explicitly derive the exact rate for finite input sequences. Thus, it is not easy to conclude which is a more suitable extractor for practical use in general. Among related work, only one, by Pae [13], compared both extractors as mentioned in Section 1.1, but it does not completely answer the question, since it analyzed the performance of both extractors only for restricted parameters, in particular, the case where each bit of input sequences occurs with probability p=1/3 and did not consider the running time. In this paper, we will perform non-asymptotic analysis for the wide range of parameters for Elias’s and Peres’s extractors, to answer the following question: which is more suitable for practical use in real-world applications? To do this, we evaluate the numerical performance of Peres’s extractor and Elias’s extractor with the RM method in terms of practical aspects including achievable rates (or redundancy) and running time with finite input sequences. Specifically, the contribution of this paper is as follows:

(i) Based on some heuristics, we derive a lower bound on the maximum redundancy of Peres’s extractor in Section 3. This result shows that the maximum redundancy of Elias’s extractor is superior to Peres’s extractor in general, if we focus only on redundancy (or rates) and we do not pay attention to the time complexity or space complexity.
(ii) By numerical analysis, we design our experiments by comparing both extractors with finite input sequences of which each bit occurs with any biased probability p∈(0,1) under the same environments in terms of practical aspects. Both extractors are implemented on a general PC and do not require any special resources, libraries, or frameworks for computation. Our implementation and results will be explained in Section 4. We calibrate our implementation by comparing the theoretical and experimental redundancy of both extractors. Afterwards, we analyze the time complexity of both extractors with respect to the bit-length of input sequences from 100 to 5000. We compare the redundancy of both extractors, and our implementation shows that Peres’s extractor is much better than Elias’s extractor under a very similar running time. As a result, Peres’s extractor would be more suitable for generating uniformly random sequences for practical use in applications.

The primary version of this paper appeared in CISS2017 [21], and this paper is an extended and full version of it. The difference between the primary version [21] and this paper is as follows: This paper contains the above result (i) in Section 3, and reports more detailed implementation results for (ii) in Section 4. In particular, we implemented and confirmed the results (ii) at a larger scale (see Section 4 for details) in addition to obtaining new figures in Section 4.1.

## 2. Preliminaries

Throughout this paper, we assume that $\log\left(\cdot \right):=\log_{2}\left(\cdot \right)$ and $\ln\left(\cdot \right):=\log_{e}\left(\cdot \right)$, and we define 0log0:=0. h(·) is the binary entropy function defined by h(p)=−plogp−(1−p)log(1−p) for p∈[0,1]. Let nk be a binomial coefficient defined by nk:=n(n−1)(n−2)⋯(n−k+1)k(k−1)(k−2)⋯1 for nonnegative integers *n* and *k*, and n0:=1 for any n≥0 (see [22] for an extension of the traditional definition of binomial coefficients). Note that nk>0 if k≤n, and nk=0 if k>n.

The first deterministic extractor was constructed by von Neumann [6] in 1951, and later improved ones were proposed by Elias [7] in 1971, and by Peres [8] in 1992. The prior work [6,7,8] considered Bernoulli source Bern(p) from which input sequences were generated, namely Bern(p) outputs i.i.d. $\left(x_{1},x_{2},\ldots ,x_{n}\right)\in {\left\{0,1\right\}}^{n}$ according to $\mathrm{Pr}(x_{i}=1)=p$ and $\mathrm{Pr}(x_{i}=0)=q=1-p$ for some unknown p∈(0,1).

A deterministic extractor A takes $\left(x_{1},x_{2},\ldots ,x_{n}\right)$ $\in {\left\{0,1\right\}}^{n}$ as input and outputs $\left(y_{1},y_{2},\ldots ,y_{\ell }\right)\in {\left\{0,1\right\}}^{\ell }$, and its average bit-length of output is denoted by $\bar{\ell }\left(n\right)$ which is a function of *n*, and defines its rate function by $r^{A}\left(p\right):=\lim_{n\to \infty }\bar{\ell }\left(n\right)/n$. Additionally, for a deterministic extractor A, we define the redundancy function by $f^{A}\left(p\right):=h\left(p\right)-r^{A}\left(p\right)$, and the maximum redundancy by $\Gamma :=\sup_{p\in (0,1)}f^{A}\left(p\right)$. Note that the above definition of redundancy functions is meaningful, since h(p) is shown to be the information-theoretic upper bound of the extractors in [7,8]. Furthermore, in this paper we define a non-asymptotic rate function $r^{A}\left(p,n\right):=\bar{\ell }\left(n\right)/n$, a non-asymptotic redundancy function $f^{A}\left(p,n\right):=h\left(p\right)-r^{A}\left(p,n\right)$, and the non-asymptotic maximum redundancy $\Gamma \left(n\right):=\sup_{p\in (0,1)}f^{A}\left(p,n\right)$, which will be used in our non-asymptotic analysis.

### 2.1. Von Neumann’s Extractor

Von Neumann’s extractor was a simple algorithm for extracting independent unbiased bits from biased bits. This algorithm divides the input sequences $\left(x_{1},x_{2},x_{3},x_{4},\ldots ,x_{n}\right)$ into the pairs (If *n* is odd, we discard the last bit.) $\left(\left(x_{1}x_{2}\right),\left(x_{3}x_{4}\right),\ldots \right)$ and maps each pair with a mapping as follows:

(1) 00↦∧,01↦0,10↦1,11↦∧,

where ∧ means no output was generated. After that, it concatenates all resulting outputs of (1). To facilitate understanding, we give an example as follows.

**Example** **1.** *Suppose that an input sequence is $(x_{1},x_{2},x_{3},$$\ldots ,x_{8})=(1,0,0,1,$0,0,1,1). Firstly, divide it into the pairs as ((1,0),(0,1),(0,0),(1,1)). Next, map each pair with the mapping (1). Finally, the extractor outputs $\left(y_{1},y_{2}\right)=\left(1,0\right)$.*

**Complexity:** Von Neumann’s extractor is efficient in the sense that both time complexity and space complexity are small such that time complexity is evaluated as O(n), and space complexity is evaluated as O(1).

**Redundancy:** Von Neumann’s extractor is not desirable, since the maximum redundancy is far from zero. In fact, the rate function $r^{\mathrm{vN}}\left(p\right)$ of von Neumann’s extractor is evaluated by $r^{\mathrm{vN}}\left(p\right)=\lim_{n\to \infty }np(1-p)/n=p\left(1-p\right)$, which is 1/4 at p=1/2 and less elsewhere. In addition, the (non-asymptotic) rate functions, (non-asymptotic) redundancy functions, and the (non-asymptotic) maximum redundancy is evaluated as follows: $f^{\mathrm{vN}}\left(p,n\right)=f^{\mathrm{vN}}\left(p\right)=h\left(p\right)-p\left(1-p\right)$, $\Gamma^{\mathrm{vN}}\left(n\right)=\Gamma^{\mathrm{vN}}=3/4$.

### 2.2. Elias’s Extractor

Elias [7] improved von Neumann’s extractor by using a block coding technique in 1971. Let N∈N (N≥2) be the block size in Elias’s extractor. For all binary sequences with bit-length *N*, partition them into N+1 sets $S_{k}$ (k=0,1,2,…,N), where $S_{k}$ consists of all the Nk sequences of length *N* which have *k* ones and N−k zeros. Here, we note that each sequence of $S_{k}$ is equiprobable, i.e., the probability $p^{k}q^{N-k}$.

We consider binary representation of the nonnegative integer |Sk|=Nk as follows: Nk=αm2m+αm−12m−1+…+α020, where m=⌊logNk⌋, $\alpha_{j}\in \left\{0,1\right\}$, and $\alpha_{m}=1$. In this case, we briefly write |Sk|=Nk=(αm,αm−1,…,α0). For each *j* (1≤j≤m) such that $\alpha_{j}=1$, we assign $2^{j}$ distinct output sequences of length *j* to $2^{j}$ distinct sequences of $S_{k}$ which have not already been assigned. If $\alpha_{0}=1$, one sequence of $S_{k}$ is assigned to ∧. In particular, since $|S_{0}\left|=\right|S_{N}|=1$, two sequences (0,0,…,0) and (1,1,…,1) are assigned to ∧. For instance, we show a procedure of Elias’s extractor in Example 2.

**Example** **2.** *Suppose that the given input sequence x=(1,0,0,1,0,0,1,1) with block size N=4, is the same as in Example 1. Firstly, we partition the set ${\left\{0,1\right\}}^{4}$ of possible input sequences into the following subsets:*

$$\begin{array}{cc} S_{0} & =\{(0,0,0,0)\},\\ S_{1} & =\{(1,0,0,0),(0,1,0,0),(0,0,1,0),(0,0,0,1)\},\\ S_{2} & =\{(0,0,1,1),(0,1,0,1),(0,1,1,0),(1,1,0,0),(1,0,1,0),(1,0,0,1)\},\\ S_{3} & =\{(1,1,1,0),(1,0,1,1),(1,1,0,1),(0,1,1,1)\},\\ S_{4} & =\{(1,1,1,1)\}. \end{array}$$

*Then, we have $|S_{0}\left|=\right|S_{4}|$$=1=\left(1\right),|S_{1}\left|=\right|S_{3}\left|=4=\left(1,0,0\right),\right|S_{2}|=6=\left(1,1,0\right)$. We consider the following assignment of output sequences:*

$$\begin{array}{ccc} & (0,0,0,0)\mapsto \wedge , & (1,1,1,1)\mapsto \wedge ,\\ & (1,0,0,0)\mapsto (0,0), & (1,1,1,0)\mapsto (0,0),\\ & (0,1,0,0)\mapsto (0,1), & (1,0,1,1)\mapsto (1,0),\\ & (0,0,1,0)\mapsto (1,0), & (1,1,0,1)\mapsto (1,1),\\ & (0,0,0,1)\mapsto (1,1), & (0,1,1,1)\mapsto (0,1),\\ & (0,0,1,1)\mapsto (0,1), & (1,0,1,0)\mapsto (1,0),\\ & (0,1,1,0)\mapsto (0,0), & (1,0,0,1)\mapsto (1,1),\\ & (0,1,0,1)\mapsto (0), & (1,1,0,0)\mapsto (1). \end{array}$$

Suppose that an input sequence x=(1,0,0,1,0,0,1,1) is given. Since the block size N=4, the sequence is divided as x=((1,0,0,1),(0,0,1,1)). By the above assignment of output sequences, the output sequence is y=((1,1)(0,1))=(1,1,0,1). Furthermore, there are several ways to assign $m_{k}$ bits to binary output sequences with the same probability that affect the output sequence *y*. Thus, the output sequence of 10010011 will not be 1101, if we use another assignment. Note that Elias’s extractor with block size N=2 is equivalent to von Neumann’s extractor, or equivalently the mapping (1). In this sense, Elias’s extractor is an extension of von Neumann’s extractor.

**Complexity:** It can be seen that the straightforward implementation of Elias’s extractor requires much space and time complexity to make a table of the assignment of output sequences, as illustrated by Example 2. Specifically, it requires exponential time and exponential memory size with respect to *N* to store all $2^{N}$ binary sequences with their assignment of output sequences. To reduce the time and space complexity of Elias’s extractor, Ryabko and Matchikina [10] proposed a method that is extended from Elias’s extractor, which we call the RM method in this paper. The RM method utilizes the enumerative encoding technique from [11] and the Schönhage–Strassen algorithm [12] for fast integer multiplication in order to compute the assignment of output sequences without making the table large. The procedure of the RM method is described as follows.

Firstly, suppose that a binary input sequence $x^{N}=\left(x_{1},x_{2},\ldots ,x_{N}\right)$ contains *k* ones and N−k zeros. Let $Num(x^{N})\geq 0$ be a number which is defined by $x^{N}$ depending on the lexicographical order set $S_{k}$. Namely, if $x^{N}$ has *k* ones, then the number Num$(x^{N})\geq 0$ is defined by

(2) Num(xN)=∑t=1NxtN−tk−∑i=1t−1xi,

where the summation is taken over all 1≤t≤N such that $x_{t}=1$, and Num$(0^{N}):=0$. Then, we calculate a binary codeword $code(x^{N})$ of $x^{N}$, which is the assignment of an output sequence of $x^{N}$ as follows:

(i) Compute Num$\left(x^{N}\right)$ in the set $S_{k}$, if $x^{N}$ contains *k* ones.
(ii) Let |Sk|=Nk=2j0+2j1+…+2jm for $0\leq j_{0}<j_{1}<\ldots <j_{m}$.
(iii) If $j_{0}=0$ and Num$(x^{N})=0$, then $code(x^{N})=\wedge$.
(iv) If $0\leq \mathrm{Num}\left(x^{N}\right)<2^{j_{0}}$, then $code(x^{N})$ is defined to be the $j_{0}$ low-order binary string of Num$\left(x^{N}\right)$.
(v) If $\sum_{s=0}^{t}2^{j_{s}}\leq \mathrm{Num}\left(x^{N}\right)<\sum_{s=0}^{t}2^{j_{s}}+2^{j_{t+1}}$ for some 0≤t≤m, then $code(x^{N})$ is defined to be the suffix consisting of the $j_{t+1}$ binary string of Num$\left(x^{N}\right)$.

**Example** **3.** *Suppose that the block size N=4, and the given input sequence is x=(1,0,0,1,0,0,1,1), which is the same as all previous examples. After that, the sequence is divided as x=((1,0,0,1),(0,0,1,1)). Next, compute Num$\left(x^{N}\right)$ following Equation (2):*

Num((1,0,0,1))=4−12+4−42−1=3,Num((0,0,1,1))=4−32+4−42−1=0.

*Then, the RM method computes code(1,0,0,1)=(1,1) and code(0,0,1,1)=(0). Finally, it outputs y=(1,1,0) by concatenating code(1,0,0,1) and code(0,0,1,1).*

The time and space complexity of Elias’s extractor with the RM method are $O(N\log^{3}N\log\log N)$ and $O(N\log^{2}N)$, respectively (see [10] for details).

**Redundancy:** Generally, the rate function and redundancy function of Elias’s extractor depend on block size *N*. For a given *n*-bit input sequence, if we take the block size equal to the length of input sequence N:=n, the rate function (or redundancy) achieve the best value. For simplicity, we assume that N=n in the following explanation. Then, the rate function $r^{E}\left(p,n\right)$ is evaluated by

(3) rE(p,n)≈1n∑k=0nnkpk(1−p)n−klognk.

Elias’s extractor takes i.i.d. with non-uniform distribution as the input, and it will output i.i.d. with uniform distribution such that its rate is given by Equation (3). Elias [7] showed that the rate function $r^{E}\left(p,n\right)$ of Elias’s extractor converges to h(p) as n→∞, or equivalently, the redundancy function $f^{E}\left(p,n\right):=h\left(p\right)-r^{E}\left(p,n\right)$ converges to zero as n→∞. More precisely, it was shown that $f^{E}\left(p,n\right)=O\left(1/n\right)$ for any fixed *p*. Therefore, for a given *n*-bit input sequence, if we set the maximum block size to be the input size, the non-asymptotic maximum redundancy $\Gamma^{E}\left(n\right)$ converges to zero not slower than 1/n.

### 2.3. Peres’s Extractor

Peres’s extractor is another method that improved the rates (or redundancy) of von Neumann’s extractor. The basic idea behind Peres’s extractor is to reuse the discarded bits in the mapping (1). In the following, we denote von Neumann’s extractor by $\Psi_{1}$. For an *n*-bit sequence $\left(x_{1},x_{2},\ldots ,x_{n}\right)$, we describe von Neumann’s extractor by $\Psi_{1}\left(x_{1},x_{2},\ldots ,x_{n}\right)$ $=(y_{1},y_{2},\ldots ,y_{\ell }),$ where $y_{i}=x_{2m_{i}-1}$ and $m_{1}<m_{2}<\cdots <m_{\ell }$ are all the indices satisfying $x_{2m_{i}-1}\neq x_{2m_{i}}$ with $m_{i}\leq n/2$. In Peres’s extractor, $\Psi_{\nu }$ (ν≥2) is defined inductively as follows: For an even *n*,

(4) $$\begin{array}{c} \Psi_{\nu }\left(x_{1},x_{2},\ldots ,x_{n}\right)=\Psi_{1}\left(x_{1},x_{2},\ldots ,x_{n}\right)*\Psi_{\nu -1}\left(u_{1},u_{2},\ldots ,u_{\frac{n}{2}}\right)*\Psi_{\nu -1}\left(v_{1},v_{2},\ldots ,v_{\frac{n}{2}-\ell }\right), \end{array}$$

where ∗ is concatenation; $u_{j}=x_{2j-1}⊕x_{2j}$ for 1≤j≤n/2; $v_{s}=x_{2i_{s}-1}$ and $i_{1}<i_{2}<\cdots <i_{\frac{n}{2}-\ell }$ are all the indices satisfying $x_{2i_{s}-1}=x_{2i_{s}}$ with $i_{s}\leq n/2$. For an odd input size *n*, $\Psi_{\nu }\left(x_{1},x_{2},\ldots ,x_{n}\right):=\Psi_{\nu }\left(x_{1},x_{2},\ldots ,x_{n-1}\right)$, i.e., the last bit is discarded and the above case of an even *n* is utilized.

Note that the number of iterations ν is at most $\left\lfloor \log n\right\rfloor$, since $\Psi_{\nu }$ for every ν≥2 is defined by $\Psi_{\nu -1}$ having an input sequence whose bit-length is at most n/2, i.e., the bit-length of both $\left(u_{1},u_{2},\ldots ,u_{\frac{n}{2}}\right)$ and $\left(v_{1},v_{2},\ldots ,v_{\frac{n}{2}-\ell }\right)$ in Equation (4) is at most n/2. Obviously, Peres’s extractor with ν=1 is the same as von Neumann’s extractor. In addition, Peres’s extractor with a large ν is considered to be an elegantly improved version of von Neumann’s extractor by utilizing a recursion mechanism.

**Example** **4.** *Suppose that an input sequence is given as x=(1,0,0,1,0,0,1,1), which is the same as all previous examples. The number of iterations satisfies $\nu \leq \left\lfloor \log8\right\rfloor =3$. Then, Peres’s extractor is executed as follows:*

$$\begin{array}{ccc} \Psi_{1}\left(x\right) & = & (1,0),\\ \Psi_{2}\left(x\right) & = & \Psi_{1}\left(x\right)*\Psi_{1}\left(1,1,0,0\right)*\Psi_{1}\left(0,1\right)=\left(1,0,0\right),\\ \Psi_{3}\left(x\right) & = & \Psi_{1}\left(x\right)*\Psi_{2}\left(1,1,0,0\right)*\Psi_{2}\left(0,1\right)\\ & = & \Psi_{1}\left(x\right)*\left(\Psi_{1}\left(1,1,0,0\right)*\Psi_{1}\left(0,0\right)*\Psi_{1}\left(1,0\right)\right)*\left(\Psi_{1}\left(0,1\right)*\Psi_{1}\left(1\right)\right)\\ & = & (1,0,1,0). \end{array}$$

**Complexity:** We denote the time complexity of $\Psi_{\nu }$ by $T_{\nu }\left(n\right)$. By Equation (4), we have

(5) $$\begin{array}{c} T_{\nu }\left(n\right)=T_{1}\left(n\right)+n/2+T_{\nu -1}\left(n/2\right)+T_{\nu -1}\left(n/2-\ell \right), \end{array}$$

and $T_{1}\left(n\right)=O\left(n\right)$ (see Section 2.1 for the time complexity of von Neumann’s extractor). From the condition (5), we obtain $T_{\nu }\left(n\right)=O\left(\nu n\right)$ for $\Psi_{\nu }$ with $1\leq \nu \leq \left\lfloor \log n\right\rfloor$. In particular, the time complexity of Peres’s extractor with the maximum iterations $\nu =\left\lfloor \log n\right\rfloor$ is evaluated as $T_{\nu }\left(n\right)=O\left(n\log n\right)$ and the space complexity is O(1).

**Redundancy:** The rate function $r_{\nu }^{P}\left(p\right)$ of Peres’s extractor can be computed inductively by the equation

(6) $$\begin{array}{cc} r_{\nu }^{P}\left(p\right) & =pq+\frac{1}{2}r_{\nu -1}^{P}\left(p^{2}+q^{2}\right)+\frac{1}{2}\left(p^{2}+q^{2}\right)r_{\nu -1}^{P}\left(\frac{p^{2}}{p^{2}+q^{2}}\right) \end{array}$$

for ν≥2, and $r_{1}^{P}\left(p\right)=pq$. Note that $r_{1}^{P}\left(p\right)$ is the rate of von Neumann’s extractor. Peres’s extractor takes i.i.d. with non-uniform distribution as input, and it will output i.i.d. with uniform distribution such that its rate is given by Equation (6) if n→∞. It is shown in [8] that $r_{\nu }^{P}\left(p\right)\leq r_{\nu +1}^{P}\left(p\right)$ for all ν∈N, p∈(0,1), and $\lim_{\nu \to \infty }r_{\nu }^{P}\left(p\right)=h\left(p\right)$ uniformly in p∈(0,1).

In other words, the above result is described in terms of redundancy as follows:

(7) $$\begin{array}{ccc} f_{\nu }^{P}\left(p\right) & = & h\left(p\right)-r_{\nu }^{P}\left(p\right)\\ & = & \frac{1}{2}f_{\nu -1}^{P}\left(p^{2}+q^{2}\right)+\frac{1}{2}\left(p^{2}+q^{2}\right)f_{\nu -1}^{P}\left(\frac{p^{2}}{p^{2}+q^{2}}\right) \end{array}$$

for ν≥2 and $f_{1}^{P}\left(p\right)=h\left(p\right)-p\left(1-p\right)$, where the above Equation (7) follows from Equation (6). Furthermore, it holds that $f_{\nu }^{P}\left(p\right)\geq f_{\nu +1}^{P}\left(p\right)$ for all ν∈N, p∈(0,1), and $\lim_{\nu \to \infty }f_{\nu }^{P}\left(p\right)=0$ uniformly in p∈(0,1). Suppose that we take the maximum $\nu =\left\lfloor \log n\right\rfloor$ and n→∞, and then, we have $\Gamma^{P}\left(n\right)=o\left(1\right)$.

In Table 1, we summarize the redundancy, time complexity and space complexity (memory size) for von Neumann’s, Elias’s, and Peres’s extractors.

## 3. Lower Bound on Redundancy of Peres’s Extractor

Although it is shown that $\Gamma^{P}\left(n\right)=o\left(1\right)$ in Peres’s extractor (i.e., $\Gamma^{P}\left(n\right)$ converges to zero as n→∞), it is not known whether $\Gamma^{P}\left(n\right)$ converges to zero rapidly or slowly. To investigate it, we analyze the non-asymptotic redundancy function $f_{\nu }^{P}\left(p,n\right)$ and non-asymptotic maximum redundancy $\Gamma^{P}\left(n\right)$. In particular, we derive a lower bound on $\Gamma^{P}\left(n\right)$ based on some heuristics.

Let $f_{\nu }^{P}\left(p\right)=h\left(p\right)-r_{\nu }^{P}\left(p\right)$ be the redundancy function for Peres’s extractor with ν iterations. Then, we first show that $f_{\nu }^{P}\left(p\right)$ is not concave in p∈(0,1) for ν≥5 as follows. The proof is given in Appendix A.

**Proposition** **1.** *The redundancy function $f_{\nu }^{P}\left(p\right)$ in Peres’s extractor with ν iterations is not concave in p∈(0,1) if ν≥5. More generally, for Peres’s extractor with ν iterations, the redundancy function $f_{\nu }^{P}\left(p\right)$ satisfies*

(8) $$\begin{array}{c} \frac{d^{2}f_{\nu }^{P}\left(\frac{1}{2}\right)}{dp^{2}}=8-\frac{4}{\ln2}-6{\left(\frac{3}{4}\right)}^{\nu -1}. \end{array}$$

*In particular, $\frac{d^{2}f_{\nu }^{P}}{dp^{2}}\left(\frac{1}{2}\right)<0$ for 1≤ν≤4 and $\frac{d^{2}f_{\nu }^{P}}{dp^{2}}\left(\frac{1}{2}\right)>0$ for ν≥5.*

**Figure A1:**
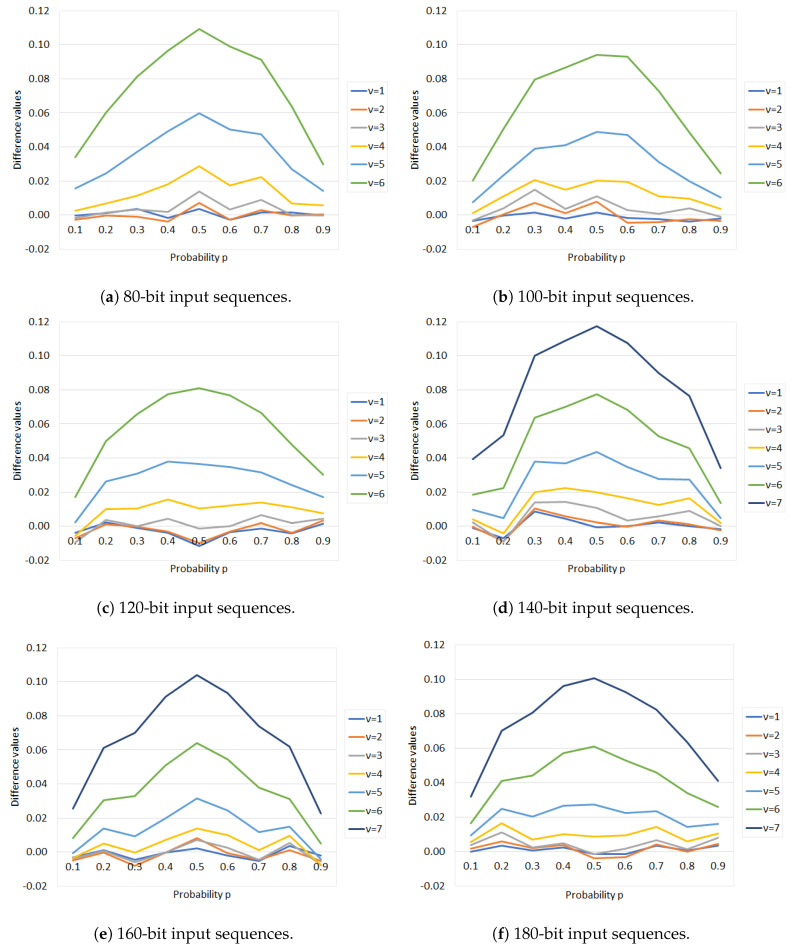

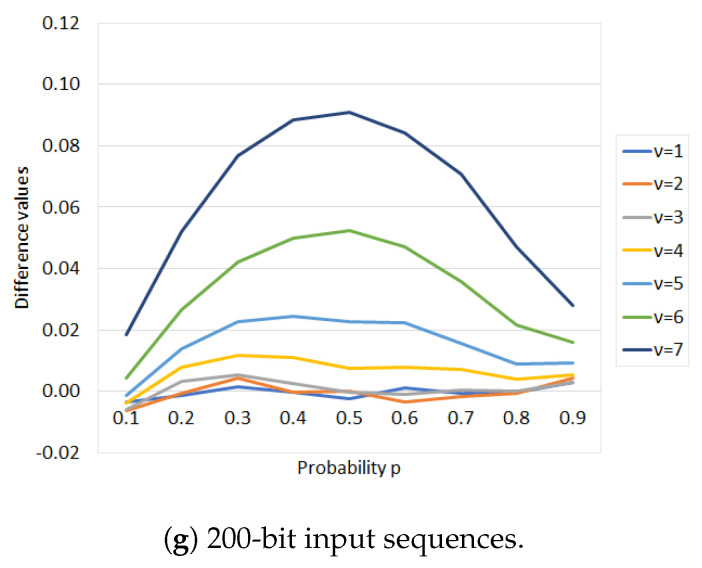
Difference values $f_{\nu }^{P}\left(p,n\right)-f_{\nu }^{P}\left(p\right)$ with n=80,100,…,200 and $1\leq \nu \leq \left\lfloor \log n\right\rfloor$.

Here, we assume that the following proposition, Proposition 2, holds true. Although it is not easy to provide proof, it seems to be true from our experimental results that are provided in Appendix B. In Figure A1 in Appendix B, we depict the difference values $f_{\nu }^{P}\left(p,n\right)-f_{\nu }^{P}\left(p\right)$ with input bit-length n=80,100,…,200 and iterations $1\leq \nu \leq \left\lfloor \log n\right\rfloor$. We note that Proposition 2 states that $f_{\left\lfloor \log n\right\rfloor }^{P}\left(p,n\right)-f_{\left\lfloor \log n\right\rfloor }^{P}\left(p\right)\geq 0$ for p∈(0,1), and we can observe that it holds true for input bit-length n=80,100,…,200 by our experimental results given in Figure A1.

**Proposition** **2** (Heuristics)**.** *Suppose $\nu =\left\lfloor \log n\right\rfloor$. Then, we have $f_{\nu }^{P}\left(p,n\right)\geq f_{\nu }^{P}\left(p\right)$, or equivalently $r_{\nu }^{P}\left(p,n\right)\leq r_{\nu }^{P}\left(p\right)$, for a sufficiently large n and any p∈(0,1).*

The following theorem shows a lower bound on $\Gamma^{P}\left(n\right)$ that is derived based on Proposition 2.

**Theorem** **1.** *Suppose that Proposition 2 holds true. Then, in Peres’s extractor with the maximum iterations $\nu =\left\lfloor \log n\right\rfloor$, we have $\Gamma^{P}\left(n\right)>1/n^{2-\log3}$. In particular, $\Gamma^{P}\left(n\right)=\omega \left(1/n\right)$.*

**Proof.**  Let *n* be a large natural number. For a natural number ν∈N with 1≤ν≤logn, we define $a_{\nu }:=r_{\nu }\left(1/2\right)$. Then, by Equation (6) we have

$$\begin{array}{c} a_{1}=\frac{1}{4},\;a_{\nu }=\frac{1}{4}+\frac{3}{4}a_{\nu -1}\;\mathrm{for}\;\nu \geq 2. \end{array}$$

By solving the above equation, we have

(9) $$\begin{array}{c} a_{\nu }=1-{\left(\frac{3}{4}\right)}^{\nu }\;\mathrm{for}\;\nu \geq 1. \end{array}$$

Thus, for $\nu =\left\lfloor \log n\right\rfloor$, we obtain

(10) $$\begin{array}{ccc} f_{\nu }^{P}\left(1/2,n\right) & \geq & f_{\nu }^{P}\left(1/2\right) \end{array}$$

(11) $$\begin{array}{cc} = & {\left(3/4\right)}^{\nu }\\ \geq & {\left(3/4\right)}^{\log n}\\ = & \frac{1}{n^{2-\log3}}, \end{array}$$

where the inequality (10) follows from Proposition 2, and the equality (11) follows from (9).

Therefore, we have

(12) $$\begin{array}{ccc} \Gamma^{P}\left(n\right) & = & \underset{p\in (0,1)}{\sup}f_{\left\lfloor \log n\right\rfloor }^{P}\left(p,n\right)\\ & > & f_{\left\lfloor \log n\right\rfloor }^{P}\left(\frac{1}{2},n\right)\\ & \geq & \frac{1}{n^{2-\log3}}, \end{array}$$

where the inequality (12) follows from Proposition 1. ☐

Theorem 1 shows that the non-asymptotic maximum redundancy $\Gamma^{P}\left(n\right)$ does converge to zero slower than 1/n. This means that Peres’s extractor is worse than Elias’s extractor in terms of the maximum redundancy, since $\Gamma^{E}\left(n\right)=O\left(1/n\right)$ if block size is set to be *n*. However, this result does not always mean that Peres’s extractor is worse than Elias’s extractor, since the time complexity and space complexity of Peres’s extractor are better than those of Elias’s extractor, as shown in Table 1. In this sense, it is not easy to conclude which extractor is superior. In the next section, from a viewpoint of practicality including running time, we compare both extractors and show that Peres’s extractor is better than Elias’s extractor by numerical analysis with various parameters.

## 4. Implementation and Numerical Analysis

In this section, we describe our experimental results of Peres’s extractor and Elias’s extractor with the RM method. We used Java language version 1.8 to implement both extractors and evaluated the performance on a desktop PC with Intel Core i3 3.70 GHz and 4 GB of RAM. Our experiments could also be performed on a general PC and do not require any special resources, libraries, or frameworks for computation. In fact, we can use other languages instead of Java language, but Java language can evaluate it on every platform without any support software. Thereby, we used Java language for implementation. To compare Peres’s extractor and Elias’s extractor with the RM method with finite input sequences in terms of non-asymptotic viewpoints, we consider the following four questions.

(1) Is theoretical redundancy the same as experimental redundancy in both extractors?
(2) Is the experimental redundancy of Elias’s extractor with the RM method better than the experimental redundancy of Peres’s extractor?
(3) What is the exact running time of both extractors?
(4) Which extractor achieves better redundancy (or rate) under the very similar running time?

To answer the above questions, we design our experiments as follows.

To answer the Questions (1) and (2), we evaluate the theoretical and experimental redundancy of Peres’s extractor and Elias’s extractor by using a pseudorandom number generation program **rand()** in MATLAB [23] to obtain biased input sequences while controlling the probability (See Section 4.1 and Section 4.2). This experiment used **rand()** to generate input sequences because we can control the probability *p* for each input sequence. Therefore, we vary the probability p=0.1,0.2,…,0.9. We show the results for a finite input sequence with 180 bits that would be used in various cryptographic algorithms. In fact, we implemented various bit-lengths of input sequences such as n=80,100,…,200 bit-length, and obtained very similar results to the case of 180-bit length (In the primary version [21], we implemented only the case of 180-bit length, and in this paper we further investigated n=80,100,…,200 bit-length.). Hence, we will describe only the input length with 180 bits, and we omit the cases of other bit-length in this paper. In addition, to investigate the efficiency of Elias’s extractor, the input size should be divided by a reasonable block size. Therefore, the 180 bit-length is also suitable, because it can be divided by many simple block-sizes 10, 20, 30, 60, 90, 180. To compute Nk in Elias’s extractor with the RM method, we consider the following:

- The Schönhage–Strassen multiplication algorithm requires $O(N^{1+\epsilon })$ which is asymptotically faster than the normal multiplication requiring $O(N^{2})$;
- To avoid multiplication, we use only the addition operation because it is simple and makes the basic operation lighter so that it can be used in various applications and environments.

Additionally, we use the recursive formula Nk=N−1k−1+N−1k for 10≤N≤180 in order to compute Nk only by additions and also by dynamic programming. To compute experimental redundancy with finite input sequences, we use 180 bit-length of inputs and generate 100 times for each probability *p*. The **rand()** will produce different sequences in every time under the same probability, thus we repeat the process to generate input sequences 100 times and calculate the average experimental redundancy. In fact, we repeated the process to generate input sequences 100, 1000, and 2000 times, but all the results on the average of experimental redundancy are almost the same, and hence, we focus on generating input sequences 100 times only (In the primary version [21], we repeated the process to generate input sequences only 100 times, and in this paper we conducted further investigations when repeating the process 1000 and 2000 times.). Next, we note that the number of iterations satisfies ν≤⌊log180⌋=7 for Peres’s extractor in Section 4.1, and we take the block size N=10,20,30,60,90,180 for Elias’s extractor with the RM method in Section 4.2. Then, we calculate the average on the redundancy function $f_{\nu }^{P}\left(p\right)$ of Peres’s extractor by using (7) and the redundancy function $f^{E}\left(p,N\right)=h\left(p\right)-r^{E}\left(p,N\right)$ of Elias’s extractor with the RM method by using (3) for each probability *p*.

To answer the Question (3), we investigate running time to extract uniformly random sequences for both extractors (See Section 4.3). Time complexity depends on the length of input sequences, and thus the probability is not a parameter in this investigation. Thereby, this experiment changes the random number generator for input sequences to RANDOM.ORG [24] to generate input sequences. This random number generator can produce a sequence that is very close to a true random number with unknown probability *p* by using the randomness of atmospheric noises. In addition, it can produce 131,072 random bits in each time. This experiment takes n=100,200,400,600,800,1000,2000,3000, 4000,5000 as the bit-length of input sequences (In the primary version [21], we took only n=100, 200,400,600,800,1000, and we further investigated longer bit-length 2000,3000,4000,5000 in this paper.). For reliability of our experiment, we repeated the process to extract unbiased random sequences 100 times for each *n*, and then calculated their average running time.

By analyzing all the results of the experiments above, we can answer the Question (4): we can compare the redundancy of both extractors under the very similar running time (see Section 4.4).

### 4.1. Analysis of the Redundancy of Peres’s Extractor

**Figure 1:**
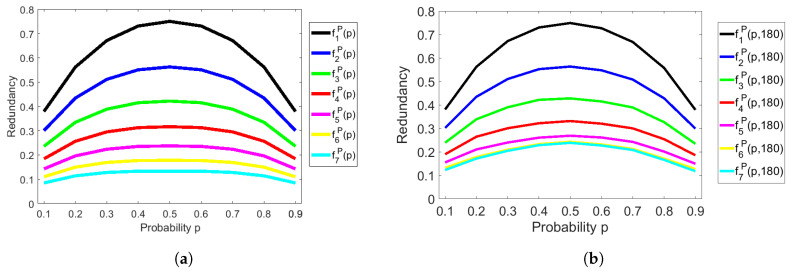
Redundancy of Peres’s extractor. (**a**) Asymptotic and theoretical estimate of redundancy by Equation (7); (**b**)Non-asymptotic and experimental estimate of redundancy with 180-bit input sequences.

In Figure 1a, we show the redundancy of Peres’s extractor from theoretical aspects, that is, we calculated the redundancy $f_{\nu }^{P}\left(p\right)$ of Peres’s extractor by using (7) with the iterations ν=1,2,…,7 and the probability p=0.1,0.2,…,0.9. We depicted the graphs of redundancy $f_{\nu }^{P}\left(p\right)$, where the *x*-axis means probability *p* and the *y*-axis means redundancy. It can be easily seen that the redundancy becomes smaller as the number of iterations becomes bigger, for all p∈(0,1). Furthermore, we showed the experimental redundancy of Peres’s extractor with 180 bit-length of input sequences in Figure 1b. As a result, the theoretical redundancy in Figure 1a is almost the same as the experimental redundancy in Figure 1b.

**Figure 2:**
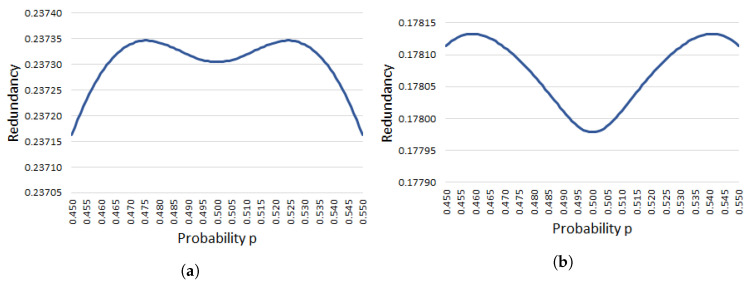
Asymptotic and theoretical estimate of the redundancy of Peres’s extractor with ν=5,6 and 0.450≤p≤0.550. (**a**) Graph of $f_{5}^{P}\left(p\right)$; (**b**) Graph of $f_{6}^{P}\left(p\right)$.

Figure 2 depicts the graphs of theoretical redundancy $f_{\nu }^{P}\left(p\right)$ with ν=5,6 around p=1/2, namely, 0.450≤p≤0.550. Both graphs support Proposition 1 from a geometric viewpoint. In addition, our experiment shows that $f_{5}^{P}\left(p\right)$ would approximately take the maximum 0.2373467 at p≈0.476 and p≈0.524, and $f_{6}^{P}\left(p\right)$ would approximately take the maximum 0.1781326 at p≈0.459 and p≈0.541.

**Figure 3:**
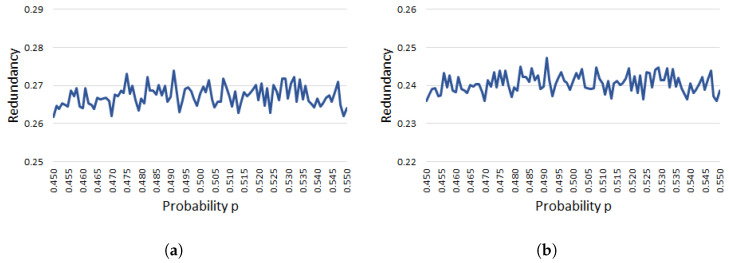
Non-asymptotic and experimental estimates on the redundancy of Peres’s extractor for 180-bit input sequences with ν=5,6 and 0.450≤p≤0.550. (**a**) Graph of $f_{5}^{P}\left(p,180\right)$; (**b**) Graph of $f_{6}^{P}\left(p,180\right)$.

Figure 3 is provided to observe the difference or similarity between $f_{\nu }^{P}\left(p\right)$ and $f_{\nu }^{P}\left(p,n\right)$ (ν≥5) for a large fixed *n*. Figure 3 shows experimental redundancy with probability 0.450≤p≤0.550 at the *x*-axis as in Figure 2. When we observe $f_{\nu }^{P}\left(p\right)$ and $f_{\nu }^{P}\left(p,n\right)$ by a rough scale, those graphs are very similar as shown in Figure 1; however, when we observe $f_{\nu }^{P}\left(p\right)$ and $f_{\nu }^{P}\left(p,n\right)$ with n=180 and ν=5,6 by a fine scale, we can see the difference between those graphs. Actually, the graphical forms of $f_{5}^{P}\left(p,180\right)$ and $f_{6}^{P}\left(p,180\right)$ are quite different from $f_{5}^{P}\left(p\right)$ and $f_{6}^{P}\left(p\right)$, respectively, as shown in Figure 2 and Figure 3, although there is the fluctuation in Figure 3 depending on our experiments. This implies that we need to analyze a non-asymptotic function $f_{\nu }^{P}\left(p,n\right)$ much more from a theoretical aspect in the future, and this analysis is also important to validate the assumption given in Proposition 2.

### 4.2. Analysis of the Redundancy of Elias’s Extractor with the RM Method

**Figure 4:**
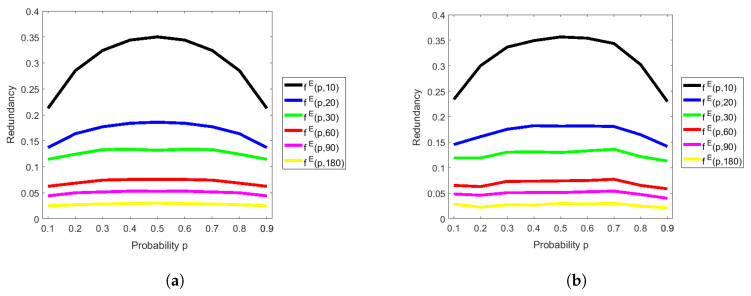
Redundancy of Elias’s extractor with the RM method. (**a**) Asymptotic and theoretical estimate of redundancy by Equation (3) and $f^{E}\left(p,n\right):=h\left(p\right)-r^{E}\left(p,n\right)$; (**b**) Non-asymptotic and experimental estimate of redundancy with 180-bit input sequences.

In Figure 4a, we show the redundancy of Elias’s extractor with the RM method from theoretical aspects, that is, we calculated the theoretical redundancy $f^{E}\left(p,N\right)=h\left(p\right)-r^{E}\left(p,N\right)$ of Elias’s extractor with the RM method by using (3) with probability p=0.1,0.2,…,0.9 and the block size N=10,20,30,60, 90,180. It can be seen that the redundancy becomes smaller as block size becomes larger, for all p∈(0,1). In spite of the fact that there is a slight difference between theoretical redundancy in Figure 4b and experimental redundancy in Figure 4a, we can say that most of them are similar.

As a result, the redundancy of Elias’s extractor with large block size is better than that of Peres’s extractor, which is an answer to our second question. Moreover, we can observe that the theoretical redundancy is almost the same as the experimental redundancy in both extractors, which is an answer to our first question. Therefore, we can rely on our implementation, and we will use this implementation for analyzing the running time in the next section.

### 4.3. Analysis of the Time Complexity of Both Extractors

**Figure 5:**
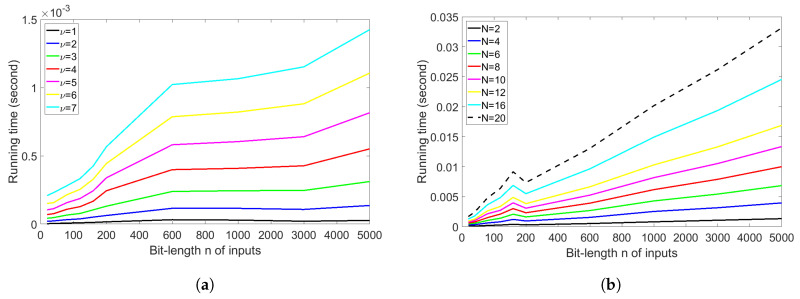
Running time. (**a**) Peres’s extractor; (**b**) Elias’s extractor with the RM method.

This section will answer the third question. In Figure 5a, we show the running time of Peres’s extractor with iterations ν=1,2,…,7 and bit-length of input sequences n=100, 200, 400, 600, 800, 1000, 2000,3000,4000,5000. We depicted the graphs of the running time, where the *x*-axis is the bit-length of input sequences and the *y*-axis is the running time in the second unit. It is clearly seen that an increase in the number of iterations leads to a large running time. The running time increases almost linearly but the slope depends on the iterations ν, as supported by a theoretical estimate of time complexity O(νn). Additionally, the running time of iterations ν=7 and the bit-length of input sequences n=5000 leads to the largest running time (1.425 milliseconds), which means that it can be used in real-world applications.

In Figure 5a, we show the running time of Elias’s extractor with the RM method with block size N=2,4,6,8,10,12,16,20. It can be seen that an increase in the block size leads to a large running time. The running time increases linearly, but the slope depends on the block size *N*, as supported by a theoretical estimate of time complexity $O(N\log^{3}N\log\log N)$. In addition, the running time with block size N=20 and bit-length of input sequences n=5000 leads to the largest running time (33.155 milliseconds), which is much larger than that of Peres’s extractor.

By comparing the running time of both extractors, the running time of Peres’s extractor is better than that of Elias’s extractor with the RM method at the same bit-length of input sequences. In the case of a long bit-length of input sequences, the difference between running time of both extractors can be seen more clearly. Therefore, we can conclude that Peres’s extractor is faster than Elias’s extractor with the RM method at the same bit-length of input sequences. On the other hand, according to the results in Section 4.1 and Section 4.2, we have seen that the redundancy of Elias’s extractor with the RM method is better than that of Peres’s extractor. Thus, we analyze the comparison of redundancy (or rate) under the very similar running time in the next section.

### 4.4. Comparison under the Very Similar Running Time

**Figure 6:**
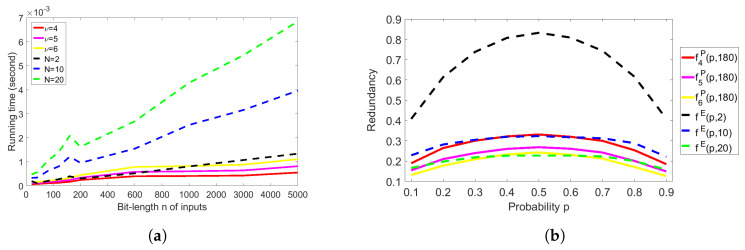
Comparison of Peres’s and Elias’s extractors. (**a**) Comparison of running time; (**b**) Comparison of redundancy for 180-bit inputs.

In all previous experiments, we have observed that the redundancy of Elias’s extractor with the RM method is better than that of Peres’s extractor; however, the time complexity of Peres’s extractor is better than that of Elias’s extractor with the RM method. Therefore, we will answer the fourth question by comparing the running time in Figure 6a and redundancy under the very similar running time in Figure 6b.

In Figure 6a, we show the comparison of the running time of Peres’s extractor with iterations ν=4,5,6 and the running time of Elias’s extractor with the RM method with block size N=2,10,20. The running time of Peres’s extractor with iterations ν=6 (the yellow line) is almost the same as the running time of Elias’s extractor with the RM method having block size N=2 (the black dashed line). Thereby, we can compare the experimental redundancy of Peres’s extractor and that of Elias’s extractor with the RM method under the very similar running time, that is, $f_{6}^{P}\left(p,180\right)$ and $f^{E}\left(p,2\right)$ in Figure 6b. It is clearly seen that $f_{6}^{P}\left(p,180\right)$ (the yellow line) is much better than $f^{E}\left(p,2\right)$ (the black dashed line), and $f_{6}^{P}\left(p,180\right)$ is close to $f^{E}\left(p,20\right)$ (the green dashed line). However, the running time of Elias’s extractor with the RM method with block size N=20 is much larger than the running time of Peres’s extractor with iterations ν=6, as seen in Figure 6a. In addition, we can observe that the redundancy $f_{4}^{P}\left(p,180\right)$ of Peres’s extractor with iterations ν=4 (the red line) is close to the redundancy $f^{E}\left(p,10\right)$ of Elias’s extractor with the RM method with block size N=10 (the blue dashed line), but the running time of Elias’s extractor with the RM method with block size N=10 is approximately 16 times larger than that of Peres’s extractor with iterations ν=4, as seen in Figure 6a (i.e., the blue dashed line and the red line). As a result, we can conclude that Peres’s extractor achieves a better rate (or redundancy) than Elias’s extractor with the RM method under the very similar running time.

## 5. Conclusions

It is known that Elias’s extractor achieves the optimal rate if the block size tends to infinity. We considered an improved version of Elias’s extractor from Ryabko and Matchikina [10] to reduce both the time complexity and space complexity. Peres’s extractor achieves the optimal rate if the length of the input and the number of iterations tend to infinity. These are the results of asymptotic analysis, but it is important and interesting to non-asymptotically analyze and compare both extractors for finite input sequences, since the resulting information will be useful in applications (e.g., cryptography) in practice.

In this paper, we evaluated the numerical performance of Peres’s extractor and Elias’s extractor with the RM method in terms of practical aspects. Firstly, we derived a lower bound on the maximum redundancy of Peres’s extractor based on some heuristics, and we showed that the maximum redundancy of Elias’s extractor (with the RM method) was superior to that of Peres’s extractor in general, if we do not pay attention to the time complexity or space complexity. We also found that $f_{\nu }^{P}\left(p\right)$ is not concave in p∈(0,1) for every ν≥5. Afterwards, we evaluated the numerical performance of Peres’s extractor and Elias’s extractor with the RM method for finite input sequences. Our implementation evaluated it on a general PC and did not require any special resources, libraries, or frameworks for computation. Our empirical results showed that Peres’s extractor is much better than Elias’s extractor for given finite input sequences under a very similar running time. As a consequence, Peres’s extractor would be more suitable to generate uniformly random sequences in practice in applications such as cryptographic systems.

## Figures and Tables

**Table 1:** Comparison of extractors.

	Redundancy Γ(n)	Time Complexity	Space Complexity
von Neumann’s extractor	3/4	O(n)	O(1)
Elias’s extractor	O(1/n)	$O(n\log^{3}n\log\log n)$	$O(n\log^{2}n)$
(with maximum block-size)	(by [7])	(by [10])	(by [10])
Peres’s extractor	O(1)	O(nlogn)	O(1)
(with maximum iterations)	(by [8])	(by [8])	(by [8])

## Appendix A. Proof of Proposition 1

First, we note that, for ν≥1,

(A1) $$\begin{array}{c} f_{\nu }^{P}\left(1/2\right)=h\left(1/2\right)-r_{\nu }^{P}\left(1/2\right)={\left(\frac{3}{4}\right)}^{\nu }, \end{array}$$

where the last equality follows from (9).

For p∈(0,1), we define $\tilde{p}:=p^{2}+{\left(1-p\right)}^{2}$ and $\hat{p}:=p^{2}/\tilde{p}$. Then, it holds that

(A2) $$\begin{array}{c} \frac{d\tilde{p}}{dp}=2\left(2p-1\right),\;\frac{d\hat{p}}{dp}=\frac{2p(1-p)}{{\tilde{p}}^{2}}. \end{array}$$

Next, for the first-order derivative of $f_{\nu }^{P}\left(p\right)$, we have

(A3) $$\begin{array}{c} \frac{df_{1}^{P}\left(p\right)}{dp}=\frac{1}{\ln2}\ln\frac{1-p}{p}+2p-1, \end{array}$$

(A4) $$\begin{array}{c} \frac{df_{\nu }^{P}\left(p\right)}{dp}=\left(2p-1\right)\left(f_{\nu -1}^{P}\left(\hat{p}\right)+\frac{df_{\nu -1}^{P}\left(\tilde{p}\right)}{dp}\right)+\frac{p(1-p)}{\tilde{p}}\frac{df_{\nu -1}^{P}\left(\hat{p}\right)}{dp}\;\mathrm{for}\;\nu \geq 2. \end{array}$$

Then, by setting p=1/2 in (A4), for ν≥2, we have

(A5) $$\begin{array}{ccc} \frac{df_{\nu }^{P}\left(1/2\right)}{dp} & = & \frac{1}{2}\frac{df_{\nu -1}^{P}\left(1/2\right)}{dp}\\ & = & {\left(\frac{1}{2}\right)}^{\nu -1}\frac{df_{1}^{P}\left(1/2\right)}{dp} \end{array}$$

(A6) $$\begin{array}{cc} = & 0, \end{array}$$

where (A5) follows from (A4), and (A6) follows from (A3).

Moreover, for the second-order derivative of $f_{\nu }^{P}\left(p\right)$, we obtain

(A7) $$\begin{array}{c} \frac{d^{2}f_{1}^{P}\left(p\right)}{dp^{2}}=-\frac{1}{\ln2}\frac{1}{p(1-p)}+2,\\ \frac{d^{2}f_{\nu }^{P}\left(p\right)}{dp^{2}}=2f_{\nu -1}^{P}\left(\hat{p}\right)+2\frac{df_{\nu -1}^{P}\left(\tilde{p}\right)}{dp}+\frac{1-2p}{\tilde{p}}\frac{df_{\nu -1}^{P}\left(\hat{p}\right)}{dp} \end{array}$$

(A8) $$\begin{array}{c} \;\;+2{\left(2p-1\right)}^{2}\frac{d^{2}f_{\nu -1}^{P}\left(\tilde{p}\right)}{dp^{2}}+\\ \;\;\frac{2p^{2}{\left(1-p\right)}^{2}}{{\tilde{p}}^{3}}\frac{d^{2}f_{\nu -1}^{P}\left(\hat{p}\right)}{dp^{2}}\;\mathrm{for}\;\nu \geq 2. \end{array}$$

Furthermore, by setting p=1/2 in (A9), for ν≥2, we have

(A9) $$\begin{array}{ccc} \frac{d^{2}f_{\nu }^{P}\left(1/2\right)}{dp^{2}} & = & 2f_{\nu -1}^{P}\left(1/2\right)+2\frac{df_{\nu -1}^{P}\left(1/2\right)}{dp}+\frac{d^{2}f_{\nu -1}^{P}\left(1/2\right)}{dp^{2}}\\ & = & 2{\left(\frac{3}{4}\right)}^{\nu -1}+\frac{d^{2}f_{\nu -1}^{P}\left(1/2\right)}{dp^{2}}, \end{array}$$

where the first equality follows from (A9), and the second equality (A9) follows from (A1) and (A6). Then, by solving Equation (A9) (ν≥2) and $\frac{d^{2}f_{1}^{P}\left(1/2\right)}{dp^{2}}=2-4/\ln2$, we obtain

(A10) $$\begin{array}{ccc} \frac{d^{2}f_{\nu }^{P}\left(1/2\right)}{dp^{2}} & = & \frac{d^{2}f_{1}^{P}\left(1/2\right)}{dp^{2}}+2\sum_{k=1}^{\nu -1}{\left(\frac{3}{4}\right)}^{k}\\ & = & 2-\frac{4}{\ln2}+6\left\{1-{\left(\frac{3}{4}\right)}^{\nu -1}\right\}\\ & = & 8-\frac{4}{\ln2}-6{\left(\frac{3}{4}\right)}^{\nu -1}. \end{array}$$

From Equation (A11), it follows that

$$\begin{array}{c} \frac{d^{2}f_{\nu }^{P}\left(1/2\right)}{dp^{2}}<0\;\mathrm{for}\;1\leq \nu \leq 4,\\ \frac{d^{2}f_{\nu }^{P}\left(1/2\right)}{dp^{2}}>0\;\mathrm{for}\;\nu \geq 5. \end{array}$$

## Appendix B. Experimental Results for Proposition 2

In this appendix, we show experimental results for Proposition 2, which support that Proposition 2 holds true. In Figure A1, we depict the difference values $f_{\nu }^{P}\left(p,n\right)-f_{\nu }^{P}\left(p\right)$ with input bit-length n=80,100,⋯,200 and iterations $1\leq \nu \leq \left\lfloor \log n\right\rfloor$. The *x*-axis is the probability p=0.1,0.2,⋯,0.9 and the *y*-axis is the difference values defined by $f_{\nu }^{P}\left(p,n\right)-f_{\nu }^{P}\left(p\right)$.

Proposition 2 states that $f_{\left\lfloor \log n\right\rfloor }^{P}\left(p,n\right)-f_{\left\lfloor \log n\right\rfloor }^{P}\left(p\right)\geq 0$ for p∈(0,1), and we can observe that it holds true for input bit-length n=80,100,⋯,200 by our experimental results given in Figure A1.
